# Microalbuminuria as a simple predictor of incident diabetes over 8 years in the Korean Genome and Epidemiology Study (KoGES)

**DOI:** 10.1038/s41598-017-15827-2

**Published:** 2017-11-13

**Authors:** Dong-Hyuk Jung, Young-Sup Byun, Yu-Jin Kwon, Gwang-Sil Kim

**Affiliations:** 1 Department of Family Medicine, Yongin Severance Hospital, Yonsei University College of Medicine, Yongin, Republic of Korea; 2 Division of Cardiology, Department of Internal Medicine, Sanggye Paik Hospital Inje University College of Medicine, Seoul, Republic of Korea

## Abstract

Microalbuminuria (MAU) is a common subclinical disease and related with cardiovascular outcome both in diabetic and non-diabetic patients. However, there is rare data about the effect of MAU on the development of diabetes. Thus, we aimed to investigate whether MAU is associated with the development of incident diabetes. A total of 3385 subjects without diabetes (1503 men and 1882 women; mean age, 53 years) who participated in the Ansung–Ansan cohort study from 2001–2002 (baseline) to 2011–2012 (fifth follow-up visit) were followed for a mean of 8 years. The prevalence of MAU at baseline was 10.8% (365 patients), and the incidence of newly developed diabetes during the follow-up period was 15.3% (56 patients) in subjects with MAU. The hazard ratio (HR) for development of diabetes was 1.43 (95% confidence interval (CI) 1.07–1.91, p-value 0.016), independent of traditional risk factors for diabetes including pre-diabetes, age, obesity, and family history. The impact of MAU on diabetes was also significant in the non-pre-diabetic population (HR 2.08, 95% CI 1.07–4.03, p-value 0.031). In conclusion, our results show that incident MAU is associated with future development of diabetes and could be an early marker for diabetes, even in the non-prediabetic population.

## Introduction

Microalbuminuria (MAU) is a common subclinical disease whose prevalence ranges from 5–7% in the general population^1^ and is about 30% in patients with hypertension (HTN)^2^. MAU is an established predictor of micro and macrovascular complications in patients with type 2 diabetes. However, recent epidemiological and experimental data have indicated that MAU is an early marker of target organ damage and is associated with all-cause mortality, CVD incidence, and progression of chronic kidney disease in non-diabetic subjects^3–5^. In addition, several studies have reported that insulin resistance or prediabetes is associated with MAU^6–8^. However, few studies have explored the effect of MAU on the development of diabetes in the non-diabetic population.

We therefore aimed to investigate whether MAU is associated with the development of incident diabetes in the general Korean population using data from the Korea Genome and Epidemiology Study (KoGES).

## Research Design and Methods

### Study Population

Study subjects were individuals who participated in the Ansung–Ansan cohort study from 2001–2002 (baseline) to 2011–2012 (fifth follow-up visit). The Ansung–Ansan cohort study is an ongoing study that began in 2001 and involves biennial follow-up examinations. A total of 10038 patients were initially enrolled in the cohort. The design and baseline characteristics of the Ansung–Ansan cohort study have been previously published^9,10^. In this study, we included 4297 subjects whose urine albumin-to-creatinine ratio (UACR) was analysed at the first visit. From these 4297 subjects, we excluded 512 who had underlying diabetes and 400 who did not attend their follow-up examinations. Finally, a total of 3385 subjects without diabetes (1503 men and 1882 women; mean age, 53 years) were enrolled in the study. Subjects were followed for a mean of 8 years. Informed consent was obtained from all study subjects. The study protocol was approved by the ethics committee of the Korean Centre for Disease Control and the Yonsei University School of Medicine Institutional Review Board.

and performed according to the principles of the Declaration of Helsinki. All data generated during this study are available.

### Clinical and biochemical parameters

Study data included medical history, physical examination, information provided by a questionnaire, anthropometric measurements, and laboratory measurement. Information on medical history, family history, current use of medications, weekly alcohol consumption, and smoking status were obtained from all participants using a standard questionnaire.

Waist circumference was measured three times at the midpoint between the bottom of the ribcage and the top of the iliac crest using a fiberglass tape measure. Blood pressure was measured in the sitting position after 5 min of rest using a standard mercury sphygmomanometer.

Body mass index (BMI) was calculated as weight divided by height squared (kg/m^2^). Physical activity was classified into the following three categories: none, irregular (≤2 episodes/week), and regular (≥3 episodes/week) exercise. One episode of exercise was defined as exercising for at least 30 min. The International Obesity Task Force (IOTF) and World Health Organization (WHO) regional office for the Western Pacific region recommend defining obesity in Asians as a BMI ≥ 25 kg/m^2^. Subsequently, the Korean Society for the Study of Obesity (KSSO) adopted this definition. Thus, subjects were classified as “generally obese” if their BMI was ≥25 kg/m^2^. Abdominal obesity was defined as a waist circumference ≥90 cm for male subjects and ≥85 cm for female subjects according to the criteria of the modified National Cholesterol Education Program’s Adult Treatment Panel III (NCEP ATP III) Asian criteria for metabolic syndrome. Collected blood samples were delivered to and analysed at a central laboratory (Seoul Clinical Laboratories, Seoul, Korea). Plasma glucose, total cholesterol, triglycerides, and HDL cholesterol levels were determined using a Hitachi 747 chemistry analyser (Hitachi, Tokyo, Japan). The LDL cholesterol level was calculated using Friedewald’s equation. The HbA1c level was measured by high-performance liquid chromatography on a Variant II instrument (BioRad Laboratories, Hercules, CA).

### Definitions of MAU, diabetes, and prediabetes

MAU was defined as an UACR of 30–300 mg/g. We excluded overt albuminuria, defined as UACR > 300 mg/g. diabetes was defined as one of the following: (1) self-reported history of diabetes; (2) HbA1c level ≥6.5%; or (3) use of an antidiabetic agent or insulin. Prediabetes was defined as a fasting plasma glucose level of 110 to 125 mg/dl or an HbA1c concentration of 5.7–6.4%.

### Statistical analyses

Data are expressed as mean and SD or as the number and percentage. Comparisons of baseline variables with respect to the presence or absence of MAU were analysed using Student’s t test for continuous variables; categorical variables were analysed using the x^2^ test. We calculated the hazard ratios (HRs) for incident diabetes using Cox proportional hazards models with potential confounding variables. We included traditional risk factors for diabetes such as age, obesity, family history of diabetes, smoking status, physical activity and prediabetes in univariate analyses. Variables with p < 0.15 in the univariate analysis were entered into multivariate analysis. We developed two different models to estimate the risk of new onset diabetes. The basic model was based only on the parameters that were easy to assess without biochemical testing. The risk factors for the incidence of diabetes in the basic model were: age, family history of diabetes and obesity. Clinical model was created by adding MAU to the basic model. For each model, the area under a receiver operating characteristic curve (AROC) was calculated. Comparison of AUC curves was calculated using Delong method. This value represents an estimate of the probability that a model assigns a higher risk to those who develop diabetes within an 8-year follow up than to those who do not. All analyses were performed using SPSS Statistics for Windows version 18.0 (IBM, Armonk, NY). For all tests, a P value < 0.05 was considered to indicate a statistically significant difference.

## Results

**Figure 1:**
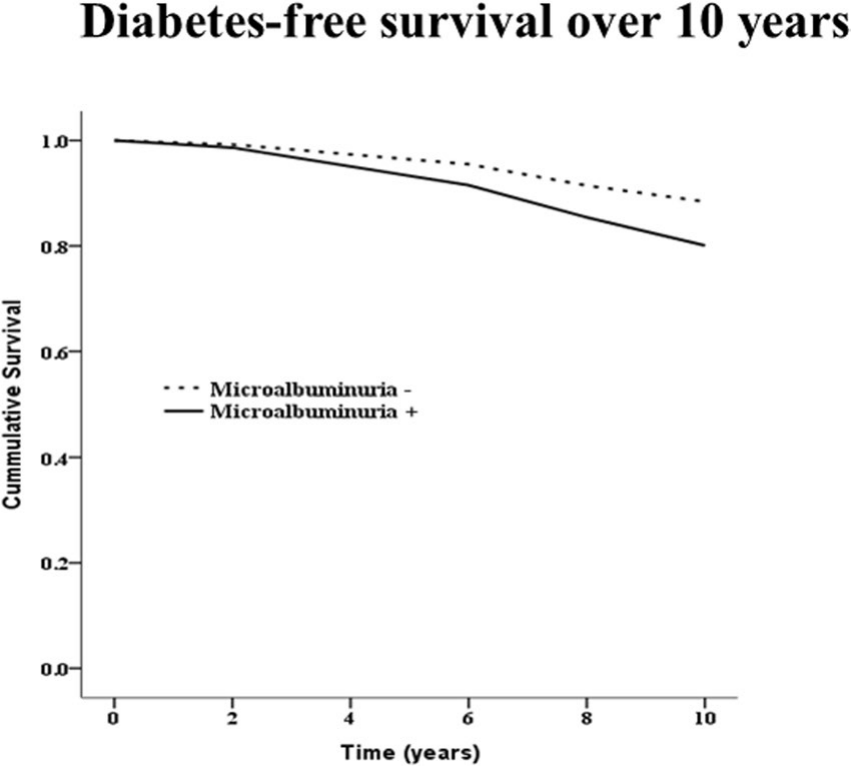
Diabetes free survival curve according to the incident microalbuminuria.

**Figure 2:**
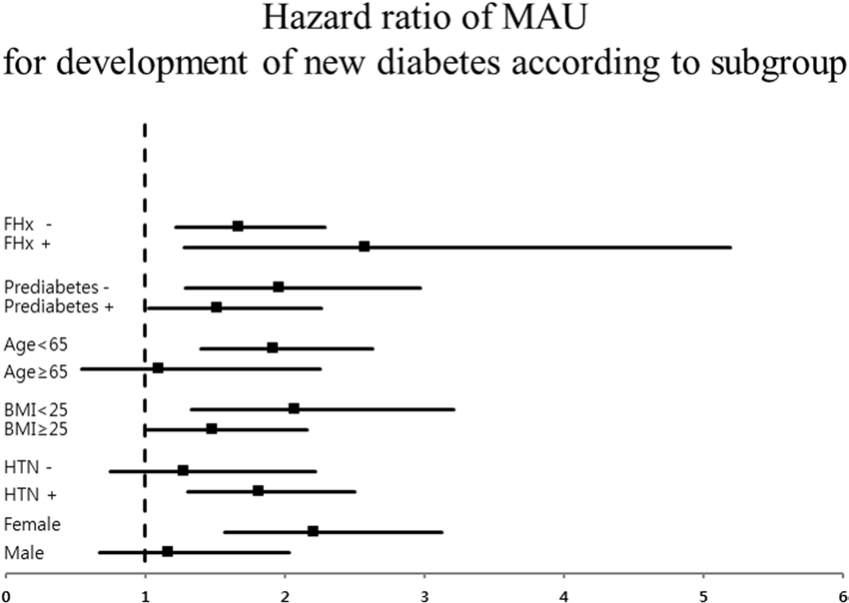
Subgroup analysis for incidental development of diabetes.

**Figure 3:**
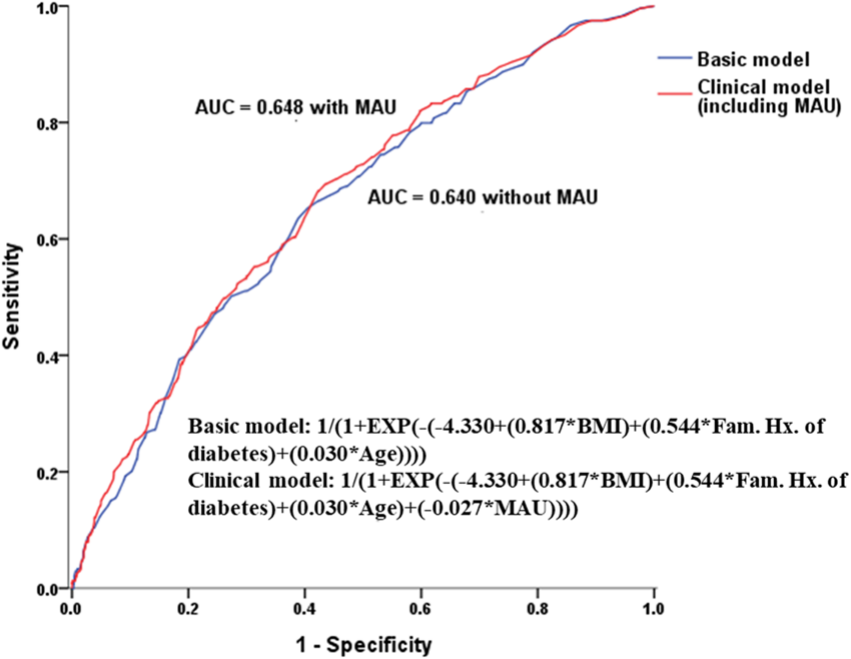
ROC curve for the prediction of diabetes in the basic and the clinical model.

The anthropometric and biochemical characteristics of the subjects are summarised according to the presence of MAU in Table 1. MAU was observed in 365 patients (10.8%) at baseline, and the mean follow up duration was approximately 8 years. The development of diabetes was increased in subjects with MAU compared with subjects without MAU after 2 years of follow up; this gap widened over time (Fig. 1). The mean age, prevalence of hypertension, and level of HbA1C increased as baseline MAU category increased. Although there was no significant difference in the presence of metabolic syndrome, the proportions of subjects with high triglycerides and impaired fasting glucose were higher in the MAU group. There were no differences in activity or family history of diabetes. Of the 365 subjects with MAU, diabetes developed in 56 (15.3%) during the 10-year follow-up period. This proportion was statistically significantly different than that in subjects without MAU. The incidences of composite cardiovascular disease and each component are shown in Table 2; no significant difference was observed according to the presence of MAU. Using a Cox proportional hazards model, we also investigated the clinical impact of MAU on the development of diabetes during the follow-up period. Participants with MAU had a higher risk for development of diabetes (hazard ratio [HR] 1.78, 95% confidence interval [CI] 1.34–2.37, p value < 0.001), as shown in Table 3. Moreover, this significant association remained after adjusting for other risk factors for diabetes including age, prediabetes, general and abdominal obesity, and family history. We next performed subgroup analysis. In the non-prediabetic group, the association was even greater (HR 2.08, 95% CI 1.07–4.03, p value 0.031), even after adjusting for confounding factors. The association between MAU and diabetes was more prominent in younger patients and in nonobese patients (Fig. 2). The AROC for the basic model and the clinical model was evaluated (Fig. 3). The AROC increased from 0.640 (95% CI, 0.609–0.671) in the basic model to 0.648 (95% CI, 0.617–0.678) in the clinical model. The addition of MAU to the basic model improved the discrimination ability; however, the AROC changed only marginally, from 0.640 to 0.648 (p value 0.099), when MAU was added to the clinical model.

**Table 1:** Baseline characteristics according to the presence of microalbuminuria.

	Microalbuminuria (+) (n = 365)	Microalbuminuria (−) (n = 3020)	P value
Age (years)	55 ± 9	52 ± 9	<0.001
Male gender	125 (34.2)	1378 (45.6)	<0.001
Hypertension	72 (19.7)	351 (11.6)	<0.001
Current smoking status	61 (16.7)	675 (22.4)	0.069
CAD	1 (0.3)	20 (0.7)	0.559
PAD	0	12 (0.4)	0.402
Stroke	5 (1.4)	33 (1.1)	0.746
SBP, mmHg	136 ± 23	123 ± 19	<0.001
DBP, mmHg	87 ± 13	80 ± 12	<0.001
Heart rate	66 ± 8	64 ± 8	<0.001
Metabolic syndrome	102 (27.9)	817 (27.1)	0.709
Triglyceride ≥ 150 mg/dl	186 (51.0)	1216 (40.3)	<0.001
Low HDL	214 (58.6)	1696 (56.2)	0.372
Abdominal obesity	101 (27.7)	911 (30.2)	0.334
Hypertension	60 (16.4)	492 (16.3)	0.940
Impaired fasting glucose	70 (19.2)	341 (11.3)	<0.001
Creatinine, mg/dl	0.8 ± 0.3	0.8 ± 0.1	0.189
HbA1_C_, %	5.64 ± 0.4	5.59 ± 0.3	0.007
Prediabetes	181 (49.6)	1252 (41.5)	0.004
BMI, kg/m^2^	24.9 ± 3.3	24.4 ± 3.1	0.003
Current alcohol	146 (40)	1345 (44.5)	0.408
Regular activity	216 (59.2)	1816 (60.1)	0.735
Family history of diabetes	37 (10.1)	294 (9.7)	0.780
Follow up duration (years)	8.3 ± 2.8	8.5 ± 2.6	0.108

Values are presented as mean ± standard deviation or n (%).

BMI, body mass index; CAD, coronary artery disease; DBP, diastolic blood pressure; HDL, high-density lipoprotein; HbA1c, haemoglobin A1c; PAD, peripheral artery disease; SBP, systolic blood pressure.

**Table 2:** Incidence of diabetes and CVD (stroke, myocardial infarction, CAD, hypertension) in non-diabetic patients during the 10-year follow up.

Clinical event	Microalbuminuria (+) (n = 365)	Microalbuminuria (−) (n = 3020)	P value
New diabetes	56 (15.3)	281 (9.3)	0.001
Composite event			
CVD	19 (5.2)	138 (4.6)	0.597
Individual events			
Stroke	7 (1.9)	55 (1.8)	0.836
Myocardial infarction	0	24 (0.8)	0.102
PAD	1 (0.3)	4 (0.1)	0.435
CAD	11 (3.0)	60 (2.0)	0.242

Values are presented as mean ± standard deviation or n (%).

CAD, coronary artery disease; CVD, cardio-vascular disease; PAD, peripheral artery disease.

**Table 3:** Multivariate analysis of the overall prevalence of diabetes and the prevalence of diabetes in non-prediabetic subjects.

	Non-adjusted		^†^Adjusted	
	Hazard ratio (95% confidence interval)	P value	Hazard ratio (95% confidence interval)	P value
**Overall population**				
Age ≥ 65	1.47 (1.10–2.0)	0.010	1.16 (0.86–1.55)	0.339
Hypertension	1.98 (1.59–2.47)	<0.001	2.87 (0.77–10.70)	0.116
Prediabetes	8.53 (6.88–10.58)	<0.001	5.98 (4.47–8.01)	<0.001
Abdominal obesity	3.61 (2.91–4.48)	<0.001	2.28 (1.79–2.90)	<0.001
Current smoking	1.41 (1.12–1.78)	0.004	1.54 (1.21–1.95)	<0.001
Family history of diabetes	1.45 (1.06–1.97)	0.020	1.58 (1.15–2.16)	0.004
BMI ≥ 25	2.07 (1.67–2.57)	<0.001	1.26 (1.00–1.60)	0.049
Microalbuminuria	1.78 (1.34–2.37)	<0.001	1.43 (1.07–1.91)	0.016
Regular activity	1.03 (0.83–1.29)	0.770		
**Non-prediabetic population**	**Hazard ratio (95% confidence interval)**	**P value**	**Hazard ratio (95% confidence interval)**	**P value**
Age ≥ 65	2.19 (1.10–4.34)	0.025	1.39 (0.69–2.82)	0.361
Hypertension	2.96 (1.73–5.06)	<0.001	2.26 (1.28–4.00)	0.005
Abdominal obesity	2.46 (1.42–4.25)	0.001	2.26 (1.26–4.04)	0.006
Current smoking	1.68 (0.95–2.94)	0.073	2.12 (1.18–3.81)	0.012
Family history of diabetes	0.92 (0.37–2.31)	0.861		
BMI ≥ 25	1.04 (0.60–1.81)	0.892		
Microalbuminuria	2.78 (1.47–5.28)	0.002	2.08 (1.07–4.03)	0.031
Regular activity	0.81 (04.7–1.37)	0.425		

BMI, body mass index.

## Discussion

In this large prospective, community-based cohort study of Korean adults, we found that MAU was associated with a 1.43-fold higher risk of development of diabetes after adjusting for several well-known risk factors for diabetes including old age, hypertension, IFG, metabolic component, and family history. The impact of MAU on diabetes was more prominent in subjects without prediabetes.

Several studies have investigated the association of MAU and insulin insensitivity or prediabetes^6–8,11^ Dutta *et al*. reported that MAU is associated with decreased reversal to normoglycemia and increased progression to diabetes with 147 pre-diabetic individuals with 3 months follow up^12^. Mykkänen *et al*. reported that MAU is associated with insulin resistance measuring fasting insulin concentration in nondiabetic subjects^13^. However, most of these were cross-sectional studies or small population with short-term follow up data; few data are available regarding whether MAU is directly associated with overt diabetes in large nondiabetic or population-based cohorts.

There is a growing interest in predictors whose abnormal levels indicate an elevated risk of development of diabetes prior to development of clinical symptoms, since regular heath checkups are becoming increasingly common and often include the acquisition of data regarding left ventricular hypertrophy, MAU, high coronary artery calcium score, and increased carotid intima-media thickness. One previous study showed that these subclinical markers are associated with an elevated risk of CVD, especially in people with metabolic syndrome and diabetes^11^. Recently, MAU was recognized as an emerging marker for cardiovascular disease in individuals with hypertension, obese individuals, and the general population, as well as acting as an emerging marker for diabetes^14–16^. The relationship between MAU and insulin insensitivity is based on several pieces of epidemiological evidence, addressing the association of kidney dysfunction with both genetic and non-genetic markers of insulin resistance^13,17–19^. Although many reports have evaluated the relationship between MAU and prediabetes, no clear consensus has yet been reached. In American group, MAU did not predict incident diabetes in pre-diabetic obese subjects^20^. Recently, two cross-sectional studies were performed in Korea. Won *et al*.^21^ reported that MAU was associated with prediabetes, while Kim *et al*. concluded that the association is probably mediated by hypertension, and MAU is not independently associated with prediabetes^8^. However, both studies were cross-sectional in nature; thus, they could not show a causal relationship between MAU and prediabetes. In addition, Won *et al*. did not include HbA1c level in the definition of diabetes, which might have led to an underestimation of the real prevalence of diabetes.

A unique aspect of our study is that we showed a direct association between MAU and incidence of diabetes using a large, nationwide cohort with a mean follow up duration of 8 years. After adjusting for traditional risk factors for diabetes including pre-diabetes and components of metabolic syndrome, the association was still significant. Since the impact of MAU was also statistically significant in subjects without prediabetes, we conclude that MAU is potentially an early marker for diabetes, similar to pre-diabetes. Although the precise mechanism of progression to the diabetes in subjects with MAU is not understood, several epidemiologic and experimental studies have reported potential mechanisms by which insulin resistance is linked to kidney dysfunction in both diabetic and non-diabetic subjects. For instance, there were epidemiologic studies showed that MAU may precede the onset of diabetes^12,22^ in non-diabetic patients. In addition, insulin insensitivity observed in non-diabetic subjects with MAU^13^ causes podocyte apoptosis and detachment from the glomerular basement membrane^23^ and insulin insensitivity also plays an important role in the development of albuminuria due to endothelial dysfunction and increased vascular permeability, even in non-diabetic patients^24^.

Although we included a large number of participants extracted by stratified random sampling with proportional allocation among the 1476 categories and tried to adjust for confounding factors, this study had some limitations. First, it was difficult to replicate our findings due to lack of availability of another cohort. Thus, lack of replication of our result is one of the major limitations. Second, urinary albumin was assessed in only a single urine specimen per participant. Since urinary albumin level can exhibit considerable intra-individual variability, the lack of repeated sampling could have missed this variation and is thus a limitation. However, there were several studies to evaluate the diagnostic performance of spot urine for the detection of MAU, since 24-hour urine collection is time and cost consuming. They showed that spot urine was a reliable measure of proteinuria in various subjects with diabetes, prediabetes, chronic kidney disease and rheumatic disease as well as in general population^25–31^. Third, MAU could transiently develop in the presence of inflammation or stress. However, we could not fully exclude such conditions, since these data were derived from a nationwide heath cohort. Therefore, another limitation is that we might have overestimated the prevalence of MAU. Fourth, this study was restricted to only Koreans. Therefore, these results might not be applicable to other ethnic groups.

In conclusion, incident MAU could be an early marker for diabetes, even in the non-prediabetic population. Thus, physicians should closely monitor all subjects and regularly measure HbA1C level. Patients should also be educated about lifestyle modifications including weight control, alcohol cessation, and regular activity.
